# Stage IV gastric adenocarcinoma with enteroblastic differentiation with 5-year relapse-free survival after D2 gastrectomy and chemotherapy: A case report

**DOI:** 10.1186/s40792-024-01921-x

**Published:** 2024-05-15

**Authors:** Hiroshi Nakayama, Tomonori Ida, Yui Hasegawa, Atsuhiko Sakamoto, Yoko Umezawa, Yuki Inaba, Hiroshi Nakada

**Affiliations:** 1 Department of Gastroenterological Surgery, Digestive Disease Center, NHO Tokyo National Hospital, 3-1-1 Takeoka, Kiyose-shi, Tokyo, 204-8431 Japan; 2 Department of Surgery, Omori Red Cross Hospital, 4-30-1 Chuo, Ota-ku, Tokyo, 143-8527 Japan; 3 Department of Gastroenterology, Omori Red Cross Hospital, 4-30-1 Chuo, Ota-ku, Tokyo, 143-8527 Japan; 4 Department of Pathology and Laboratory Medicine, Omori Red Cross Hospital, 4-30-1 Chuo, Ota-ku, Tokyo, 143-8527 Japan; 5 Department of Pathology, Fukushima Medical University Hospital, 1 Hikariga-Oka, Fukushima, 960-1295 Japan

**Keywords:** Gastric adenocarcinoma with enteroblastic differentiation, Spalt-like transcription factor 4, Stage IV, M1, Peritoneal cytology

## Abstract

**Background:**

Gastric adenocarcinoma with enteroblastic differentiation (GACED), a rare subtype of gastric cancer, is associated with a more aggressive behavior than conventional gastric adenocarcinomas. We report a rare case of stage IV GACED treated with D2 gastrectomy and postoperative chemotherapy.

**Case presentation:**

A 39-year-old woman with acute upper abdominal pain immediately underwent surgery for gastric perforation. Afterward she was diagnosed with adenocarcinoma of the pylorus. D2 gastrectomy was performed and the final pathological diagnosis was stage IV GACED with positive peritoneal cytology. Postoperative chemotherapy was initiated with S1 plus oxaliplatin for 1 year, which was ceased thereafter to enhance her quality of life. The patient survived more than 5 years without relapse after gastrectomy.

**Conclusions:**

Stage IV GACED, determined by positive spalt-like transcription factor 4, can be successfully treated with surgery and chemotherapy.

## Background

Gastric adenocarcinoma with enteroblastic differentiation (GACED), a rare subtype of gastric cancer [1–4], is more aggressive than conventional gastric adenocarcinoma [5, 6]. It is unclear whether GACED chemotherapy is as effective as that for conventional gastric adenocarcinomas due to its rarity. Positive peritoneal lavage cytology (CY1) is an important prognostic factor [7]. According to the tumor, node, metastasis (TNM) classification of the Union for International Cancer Control (UICC), the American Joint Committee on Cancer (AJCC) staging system for stomach cancer, and the Japanese Classification of Gastric Carcinoma, CY1 is classified as M1, pathological stage IV [2, 8–10]. In patients with CY1, surgery alone has resulted in death within 5 years, whereas postoperative chemotherapy has been reported to increase 5-year overall survival (OS) to approximately 20% [11, 12]. This report presents a case of advanced GACED with CY1 that was successfully treated with D2 gastrectomy and postoperative chemotherapy.

## Case presentation

**Fig. 1:**
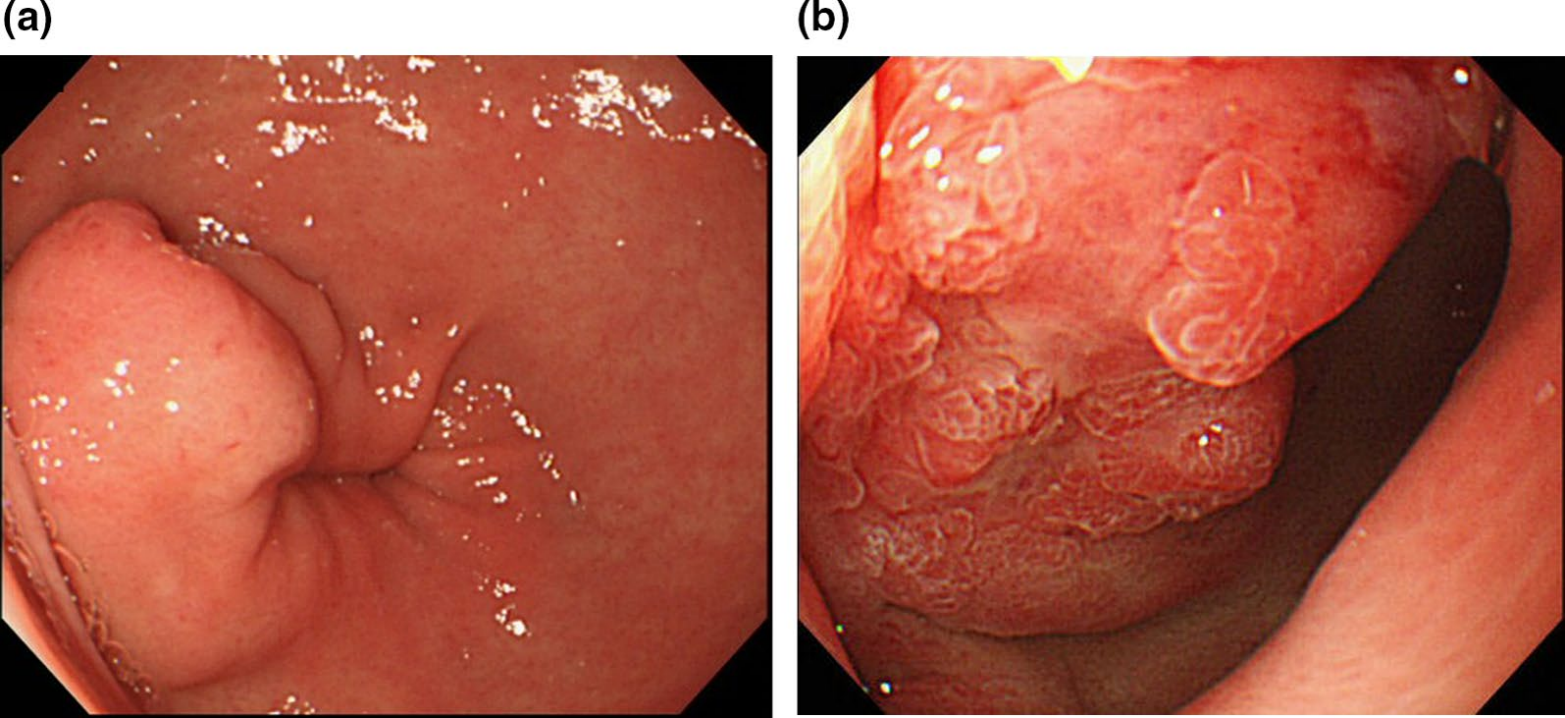
Upper gastrointestinal endoscopic features. **a** Submucosal tumor-like lesion on the anterior half of the pylorus. **b** When the scope was extended, irregular nodules with an amorphous surface were observed. Biopsy confirmed adenocarcinoma

**Fig. 2:**
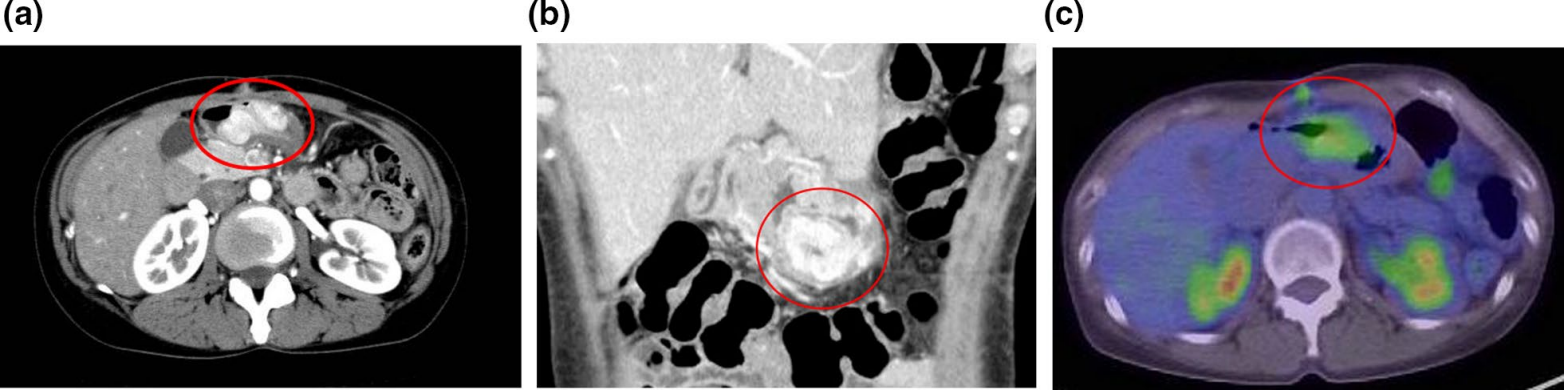
Contrast-enhanced computed tomography (**a**, **b**), positron emission, and computed tomography (**c**). The red line encircles the gastric tumor. **a**, **b** The tumor was mainly located in the gastric wall. The fat layer between the stomach and pancreatic head was intact on the dorsal side. **c** The tumor showed uptake (maximum standardized uptake value: 4.47), whereas no other metastasis was suggested

**Fig. 3:**
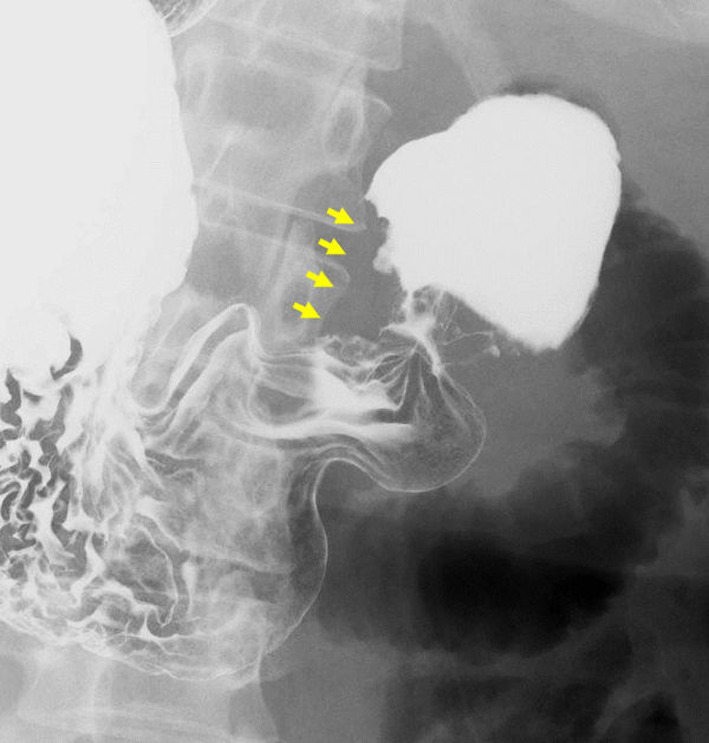
Gastroduodenal barium examination. An irregularly shaped shadow defect (yellow arrow) is shown on the pylorus, involving 1 cm of first portion of the duodenum

A 39-year-old woman was admitted to our hospital with acute upper abdominal pain in September 201X. She underwent laparotomy for gastric perforation. The procedure involved simple closure with omental coverage. Anterior wall of the pylorus, 5 mm in diameter, was perforated. The tissues around the perforation site hardened because of associated inflammation. Moreover, no findings, such as a mass, serosal irregularity, or disseminated nodules, indicated tumor presence. After surgery, the patient was diagnosed with adenocarcinoma of the pylorus by endoscopic biopsy. The endoscopic findings revealed a submucosal tumor with partial exposure to the surface mucosa (Fig. 1). Figure 2 shows contrast-enhanced computed tomography, ^18^F-fluorodeoxyglucose positron emission, and computed tomography. Figure 3 depicts the barium examination. The findings suggested that the adenocarcinoma expanded in the submucosal gastric wall.

Gastric adenocarcinoma usually does not present a submucosal tumor morphologically because it is an epithelial tumor. A heterotopic pancreas is a well-known submucosal lesion of the gastric antrum. Thus, we considered the possibility that adenocarcinoma from a heterotopic pancreas invaded the gastric lumen. Table 1 presents the blood chemistry tumor markers. Only SPan-1 was above normal. The preoperative diagnosis of this patient was a heterotopic pancreatic carcinoma of the stomach; however, the definitive diagnosis of heterotopic pancreatic carcinoma requires the pathological examination of the resected specimen to meet the three conditions proposed by Guillou et al. [13]. Adenocarcinoma of the stomach, which would be c-stage IIB (cT3N0M0), was also considered a differential diagnosis [2, 4, 9].

**Table 1:** Preoperative tumor markers

CEA	1.9 ng/mL (< 5.0)
CA19-9	7.0 U/mL (< 37.0)
**Span-1**	**220** U/mL (< 30.0)
DUPAN-2	120 U/mL (< 150.0)

Data in parentheses denote the normal values

Surgery was performed to determine the original organ and prevent pyloric obstruction. Three months after the first procedure, a distal gastrectomy with systematic D2 lymph node dissection (Roux-en-Y reconstruction) was performed. The surgical findings indicated that the tumor was in the anterior wall of the antrum and pylorus of the stomach. Additionally, the greater omentum covered the perforation site, but the serosal surface of the tumor was smooth. No macroscopic tumor dissemination was observed. A heterotopic pancreas approximately 1 cm in diameter was observed on the posterior side of the tumor, which was also dissected en bloc. Our intraoperative diagnosis was heterotopic pancreatic carcinoma of the stomach, invading the stomach and first portion of the duodenum. During laparotomy, peritoneal lavage cytology was performed.

**Fig. 4:**
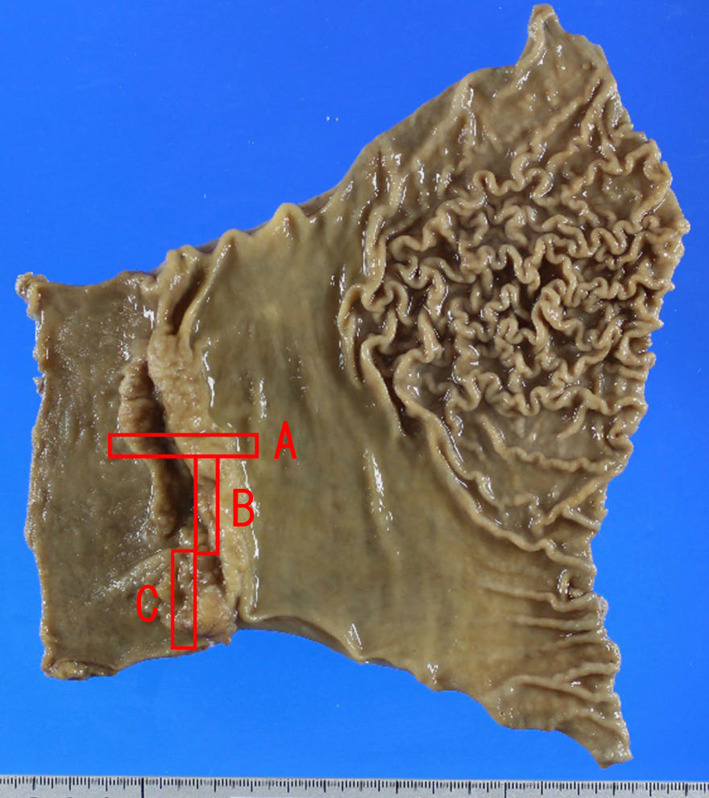
Resected gastric specimen. The tumor was 88 × 32 mm and occupied 80% of the pyloric circumference. The tumor destroyed most of the normal structure of the pyloric ring. Sections marked with red squares are detailed in Figs. 5 and 6

**Fig. 5:**
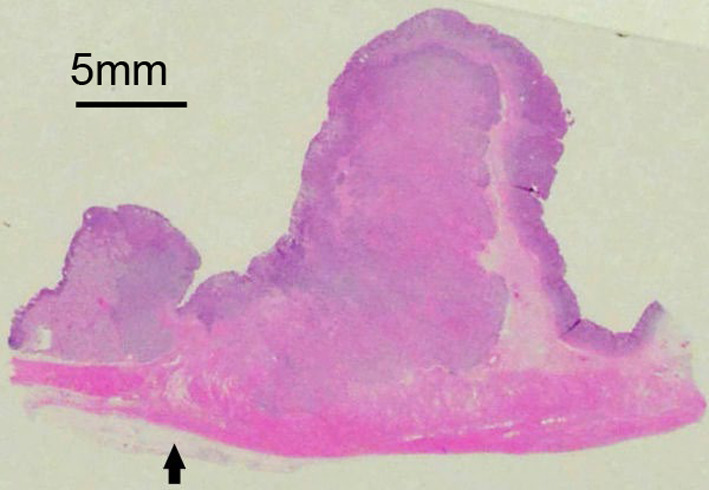
Magnified view of section A in Fig. 4 (hematoxylin and eosin). The tumor has an ulcer in its center, forming a high bank at the oral front. The original location of the pylorus ring, which is suspected from the structure of muscularis propria of the stomach and duodenum, is marked by an arrow

**Fig. 6:**
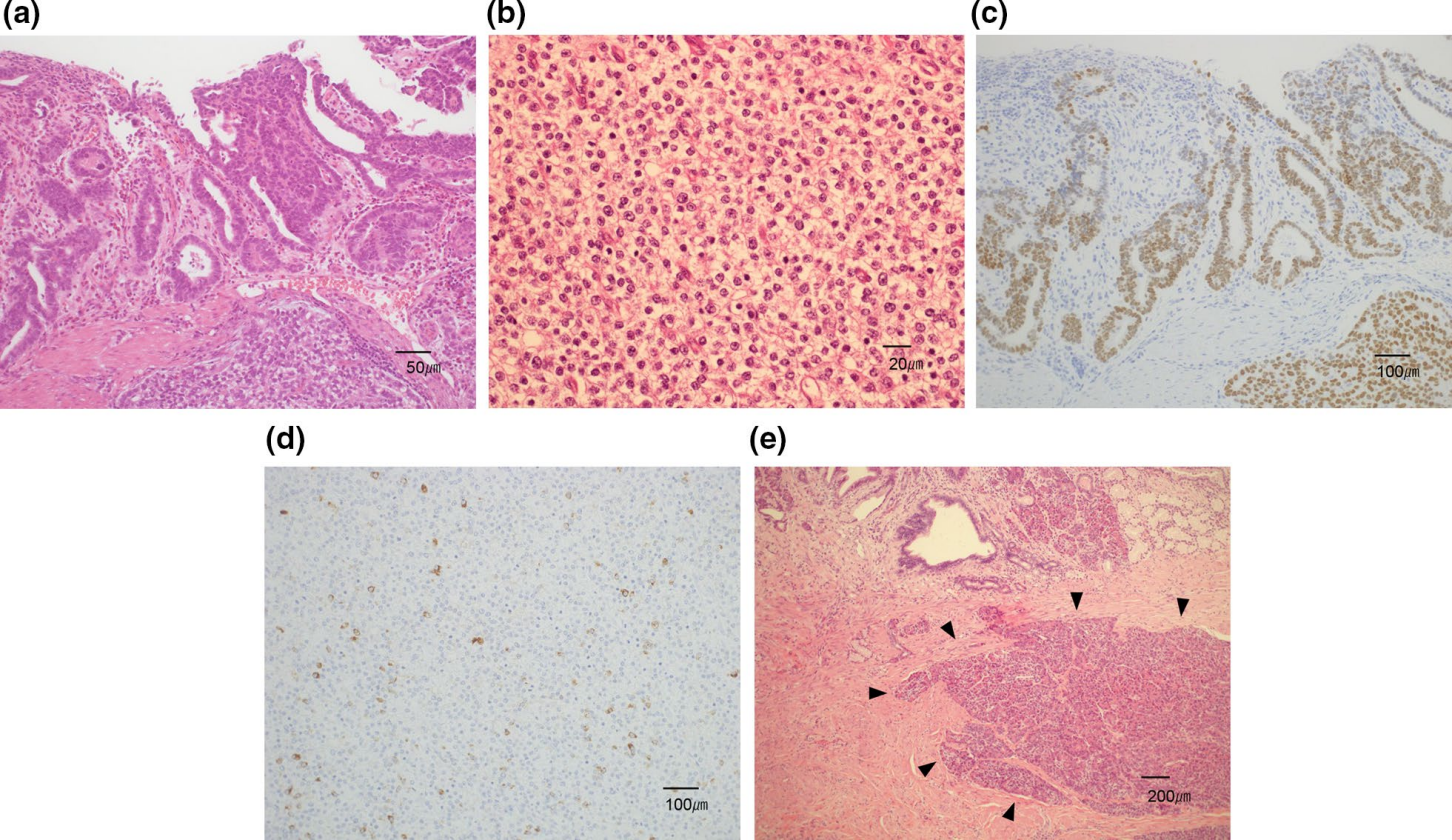
Microscopic view of the tumor. **a** Mucosal lesion of a tubular adenocarcinoma in section B of Fig. 4. **b** In the deeper layer, the tumor cells have clear cytoplasm and form solid components. **c** Both surface and deeper lesions are spalt-like transcription factor 4 (SALL4)-positive (immunohistochemistry). **d** Alpha-fetoprotein (AFP) is sporadically positive (immunohistochemistry). **e** Heterotopic pancreas in the stomach submucosa in section C of Fig. 4. The heterotopic pancreas (marked by arrows) has no atypical cells or invasive changes, although it is adjacent to the adenocarcinoma

**Fig. 7:**
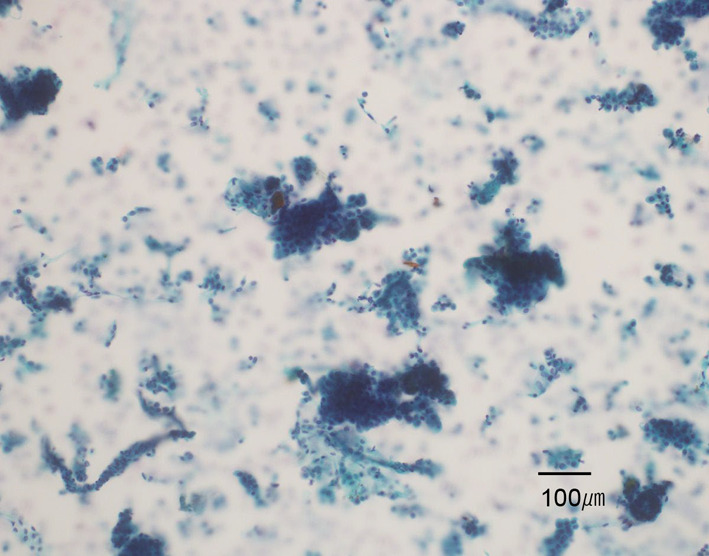
Peritoneal lavage cytology. Many papillary cancer cell clusters were identified

Figures 4, 5, 6 show the pathological findings. The pathological findings revealed tubular mucosal adenocarcinoma. The tumor cells had clear cytoplasm and were diffusely spalt-like transcription factor 4 (SALL4)-positive. No atypical findings were observed in the heterotopic pancreas. Based on these findings, the patient was finally diagnosed with GACED. Furthermore, peritoneal lavage cytology was positive (Fig. 7). According to the classification of the Japanese Gastric Cancer Association, pathological findings are described as follows: LD, LessAntPost. Type 3, 88 × 32 mm, adenocarcinoma with enteroblastic differentiation, pT3, INFb, Ly1a, v0, pN0 (0/25), pPM0 (103 mm), pDM0 (34 mm), M1 (CY1), and stage IV [2, 8]. The TNM classification of AJCC classified the tumor as pT3pN0M1, pStage IV [9].

The postoperative course was good. Postoperative chemotherapy with S1 plus oxaliplatin (SOX) (100 mg/m^2^/day S-1 for 2 weeks with 100 mg/m^2^ oxaliplatin on day 1, every 3 weeks) was started 47 days after gastrectomy. A total of 14 cycles were administered without severe adverse effects. One year after the gastrectomy, a laparotomy was performed to treat an incisional hernia. At that time, no macroscopic peritoneal metastasis was found and second peritoneal lavage cytology was negative for cancer. Chemotherapy was discontinued after a discussion with the medical team. Five and a half years after gastrectomy, the patient was well with no recurrence.

## Discussion

Matsunou et al. first reported that some alpha-fetoprotein (AFP)-producing gastric carcinomas had clear cytoplasm and morphologically resembled the fetal gut epithelium [14]. Glypican-3 (GPC3), an oncofetal protein, is expressed in AFP-producing hepatoid adenocarcinoma of the stomach [15]. The expression of another oncofetal protein, SALL4, has been reported to be a marker of fetal gut differentiation in hepatoid gastric carcinoma [1]. Yamazawa et al. investigated 386 gastric adenocarcinomas using primitive phenotype markers such as AFP, GPC3, and SALL4. Diffuse SALL4 staining was observed in 36 cases (10.2%), whereas AFP was positive in 10 cases (2.8%) [5]. Additionally, Akazawa et al. investigated 2,273 primary gastric cancers and determined GACED by immunohistochemistry staining of AFP, GPC3, and SALL4. GACED was detected in 51 cases (2.2%), whereas SALL4 was positive in 41 cases (1.8%), and AFP was positive in 16 cases (0.7%). The SALL4-positive phenotype was frequently observed in the AFP-positive group [3]. GACED has been adopted as a new subtype since the 15th edition of the Japanese Classification of Gastric Carcinoma [2]. It has also been reported as a related entity of hepatoid adenocarcinoma in the 5th edition of the WHO classification [4]. This subtype is more aggressive than conventional gastric adenocarcinoma, with a higher incidence of vessel involvement and hepatic metastasis [5, 6, 16].

Stage IV GACED is expected to have a poor prognosis. A search of PubMed and Google Scholar case reports in English and Ichushi-Web (NPO Japan Medical Abstracts Society) in Japanese was conducted using the key terms “gastric cancer”, “adenocarcinoma with enteroblastic differentiation”, and “M1” or “stage IV.” Every hit publication was investigated, but none reported a 5-year survival. This case report is the first of a patient with stage IV SALL4-positive GACED who survived for more than 5 years without relapse after gastrectomy.

This case was first admitted with gastric perforation and was later diagnosed with adenocarcinoma of the pylorus and duodenum. An endoscopic examination of the antrum revealed a lesion resembling a submucosal tumor. However, the mucosal lesion could be recognized over the stenosis. This finding was unusual for a common type of gastric adenocarcinoma. Moreover, SPan-1, not CEA or CA19-9, was elevated. Therefore, we hypothesize that adenocarcinoma arises from the heterotopic pancreas of the stomach rather than being the usual gastric or duodenal adenocarcinoma. Pathological confirmation of tubular adenocarcinoma of the gastric mucosa and no atypical findings in the heterotopic pancreas revealed that this tumor originated from the stomach. It is not rare that GACED is associated with intestinal-type adenocarcinoma [1, 4].

In the TNM classification of the UICC, the AJCC, and the Japanese Classification of Gastric Carcinoma, CY1 indicates M1 and pathological stage IV [2, 9, 10]. In this setting, survival is dismal without postoperative chemotherapy, as CY + patients die within 3 years without chemotherapy [7].

Yamada et al. showed that SOX was non-inferior to cisplatin plus S1 (CS) in first-line chemotherapy for curatively unresectable gastric cancer (JapicCTI-101021). This G-SOX trial included patients with peritoneal metastasis (approximately 19%). A subgroup analysis showed significantly longer OS in these patients [17]. Furthermore, the ARTIST2 trial compared postoperative adjuvant chemotherapy with single-agent S1 and SOX. In patients with D2-resected, stage II or III, node-positive gastric cancer, SOX showed significantly better 3-year disease-free survival than S1 [18].

In a multicenter retrospective study, Yamaguchi et al. investigated the efficacy of chemotherapy in patients with CY1 or P1a (positive localized peritoneal metastasis) with D2-resected gastric cancer. After surgery, the patients were without macroscopically visible disease, similar to our case. Patients were divided into four groups based on postoperative chemotherapy: S1 monotherapy (S1), CS, other regimens (Others), and no chemotherapy (No-Cx). The No-Cx group showed a 5-year relapse-free survival (RFS) rate of 0%, whereas the S1, CS, and Others groups showed 5-year rates of 19.3%, 17.0%, and 17.4%, respectively. RFS was significantly worse in the No-Cx group, and no statistically significant differences in RFS were observed between the CS group, Others group, and S1 monotherapy group. They concluded that CS has no merit over S1 monotherapy in patients with CY1 or P1a with D2-resected gastric cancer [11]. However, based on the results of the G-SOX trial, it cannot be denied that SOX is a candidate as a treatment regimen for these patients.

In this case, chemotherapy was initiated before Yamaguchi’s publication. SOX was selected and administered for 1 year. Practically, chemotherapy is continued in patients with stage IV gastric cancer as long as its effectiveness and adverse events are permissive. It is difficult to determine if chemotherapy can be stopped when the patient is well and without recurrence after a certain duration of chemotherapy. Mezhir et al. reported 48 follow-up laparoscopy cases in 291 CY1 patients. Those converted to negative cytology showed prolonged disease-specific survival (DSS) compared with the persistently positive group, even though the converted group had no 5-year DSS [19]. Fukagawa reviewed the role of staging laparoscopy before gastrectomy. After staging laparoscopy and neoadjuvant chemotherapy, second-look laparoscopy may be useful to determine if conversion surgery applies to the patient.

Conversely, a false negative (CY0 at staging laparoscopy turned to CY1 at gastrectomy several weeks later) exists in more than 10% of cases at the initial staging laparoscopy [20]. These facts indicate that turning to negative cytology is not equivalent to a completely cured status and long-term survival. Therefore, turned-negative peritoneal cytology and confirmative follow-up laparoscopy are insufficient to recommend the discontinuation of chemotherapy so far. For individualized care for patients with advanced cancer, the conversation between a patient and the medical team regarding the prognosis, available medical interventions, alternatives, and the patient’s preferences is critically important [21]. Pausing chemotherapy is one choice that enhances the quality of life during treatment for a patient with stable disease. In our case, we had an opportunity for laparotomy after a year of chemotherapy and confirmed that peritoneal lavage cytology was negative. The risks and benefits of further chemotherapy were discussed with the patient according to the “shared decision-making process” and chemotherapy was discontinued.

We planned gastrectomy if the lesion was resectable, independent of the result of peritoneal cytology, to determine the origin of the adenocarcinoma and to avoid pyloric obstruction due to lesion progression. In the JCOG 0501 trial, patients without a macroscopic unresectable factor confirmed by staging laparoscopy were assigned to surgery and postoperative S1 monotherapy (Arm A) and neoadjuvant chemotherapy by CS followed by D2 gastrectomy plus adjuvant chemotherapy with S1 (Arm B). Thus, Arm B failed to demonstrate OS and disease-free survival benefits. Therefore, they concluded that D2 surgery followed by adjuvant chemotherapy is standard treatment [22].

Data on the oncological behavior and prognosis of GACED, a rather new histological entity in a special type of gastric adenocarcinoma, are insufficient. This case suggests that advanced GACED can be treated by surgery and chemotherapy, like common-type gastric adenocarcinoma. Further accumulation of cases will enable better GACED management.

## Conclusions

We report a case of stage IV GACED with CY1, which is expected to have a poor prognosis. D2 gastrectomy and postoperative chemotherapy with SOX for 14 cycles resulted in negative peritoneal cytology. The patient survived for more than 5 years after gastrectomy without relapse. Although GACED is considered an aggressive subtype of gastric adenocarcinoma, surgery combined with chemotherapy has the potential to improve its prognosis.

## Data Availability

Data sharing does not apply to this article because no datasets were generated or analyzed.

## Abbreviations

GACED: Gastric adenocarcinoma with enteroblastic differentiation
CY1: Positive peritoneal lavage cytology
TNM: Tumor, node, metastasis
UICC: The Union for International Cancer Control
AJCC: The American Joint Committee on Cancer
OS: Overall survival
SALL4: Spalt-like transcription factor 4
SOX: S1 plus oxaliplatin
AFP: Alpha-fetoprotein
GPC3: Glypican-3
CS: Cisplatin plus S1
RFS: Relapse-free survival
DSS: Disease-specific survival
